# Efficacy of intravesical Bacillus Calmette-Guérin therapy against tumor immune escape in an orthotopic model of bladder cancer

**DOI:** 10.3892/etm.2014.2060

**Published:** 2014-11-11

**Authors:** PENG HUANG, CHAO MA, PENG XU, KAI GUO, ABAI XU, CHUNXIAO LIU

**Affiliations:** Department of Urology, Zhujiang Hospital, Southern Medical University, Guangzhou, Guangdong 510282, P.R. China

**Keywords:** Bacillus Calmette-Guérin, bladder cancer, immune-escape, myeloid-derived suppressor cells

## Abstract

The aim of this study was to evaluate the antitumor immune response of the Bacillus Calmette-Guérin (BCG) in an orthotopic bladder cancer model. The murine bladder cancer cell line MBT-2 was transurethrally implanted in the bladder of syngeneic female C3H/He mice. The animals were randomly divided into three treatment groups: Phosphate-buffered saline (PBS), low-dose BCG and high-dose BCG. The analyses of luciferin-stained tumor images 28 days after each treatment showed significant tumor growth inhibition in the high-dose group in comparison with that in the low-dose- or PBS-treated groups. In addition, the percentage of myeloid-derived suppressor cells in the high-dose group was significantly suppressed in comparison with that in the PBS and low-dose agent treatment groups. These findings are notable in terms of the clinical evaluations of this therapy for patients with bladder cancer. The outcomes of this study also provide important implications regarding antitumor immune responses in human cancer.

## Introduction

Urothelial cancer of the bladder is the fourth most common malignancy diagnosed in the USA. It is estimated that ~74,690 patients were diagnosed in 2014 (1,2). Among the cases of urothelial cancer of the bladder, ~75% are superficial bladder cancer with low-grade, noninvasive or superficial tumors confined to the mucosa. Patients with superficial bladder cancer are at high risk of relapse following surgery with concomitant radiotherapy and chemotherapy (3,4). The goal of treatment for these types of cancer is reducing tumor recurrence and preventing tumor progression, which would require additional aggressive therapies. Intravesical immunotherapy with Bacillus Calmette-Guérin (BCG) is an effective adjuvant therapy for superficial bladder cancer (5). The anticancer effect of BCG in the treatment of bladder cancer involves a complex local immune response, including the activation of B, T and natural killer cells induced by multiple cytokines, for example interleukin (IL)-1, -6 and -8 and granulocyte-macrophage colony-stimulating factor (6,7).

Although a clinical evaluation of the effectiveness of BCG indicates that it can induce robust immune responses against tumor antigens in patients with bladder cancer, the clinical benefits of BCG have been limited. A previous study identified cells of myeloid origin that are potent suppressors of tumor immunity and therefore represent a significant obstacle against tumor immunotherapy (8). Myeloid-derived suppressor cells (MDSCs) have been shown to accumulate at tumor sites as well as in the blood, lymph nodes and bone marrow in the majority of patients and experimental animals with cancer, and inhibit both adaptive and innate immunity (9,10). In mice, MDSCs are uniformly characterized by the expression of cell surface molecules detected by antibodies against Gr1 and cluster of differentiation (CD)11b. MDSCs act to suppress antitumor immunity through a number of diverse mechanisms. T-cell activation is suppressed by the production of reactive oxygen species and arginase, the nitration of the T-cell receptor and the induction of regulatory T cells (Tregs). Innate immunity is impaired by the increase in the production of IL-10 by MDSCs, the downregulation of macrophage-produced IL-12 and the inhibition of natural killer cell cytotoxicity (11). In the present study, the antitumor immune suppressive activity of intravesically administered BCG in an immunocompetent mouse model was investigated. Ideally, new therapeutic strategies should be tested rigorously in a relevant animal model.

## Materials and methods

### Animals

A total of 21 eight-week-old female C3H/HeN mice were obtained from Guangdong Provincial Research Center for Laboratory Animal Medicine (Foshan, China). The mice were maintained at the Animal Center of Southern Medical University (Guangzhou, China) in a specific pathogen-free environment with food and water provided *ad libitum*. The animals were housed and handled in accordance with the Southern Medical University Animal Research Committee Guidelines.

### Cell line and reagents

The murine bladder cancer cell line MBT-2 was provided by the American Type Culture Collection (Rockville, MD, USA). The MBT-2 cells were maintained in RPMI-1640 medium supplemented with 10% fetal bovine serum. The cells were cultured at 37°C in a 5% CO_2_ atmosphere and routinely passaged by trypsin-EDTA treatment in 100-cm^2^ flasks containing BCG (81 mg; Connaught substrain, ImmuCyst, Nihou Kayaku, Inc., Tokyo, Japan), and phosphate-buffered saline (PBS) for *in vivo* studies.

### In vivo effects in the murine bladder cancer models

To establish the orthotopic bladder cancer tumors, the mice were anesthetized by the intraperitoneal (i.p.) administration of ketamine/xylazine solution at dose of 0.1 ml/10 g body weight (K113; Sigma-Aldrich Japan G.K., Tokyo, Japan). A 24-gauge Teflon intravenous catheter was subsequently inserted through the urethra into the bladder using an inert lubricant. In order to prepare the bladder for tumor implantation, a brief acid exposure, followed by alkaline neutralization, promoted a chemical lesion on the bladder wall, performed by the intravesical instillation of 8 μl 1 MOI silver nitrate. This led to the formation of an adequate and controlled diffuse bladder wall lesion. After 15 sec, the content was washed out by transurethral infusion of PBS. The first catheter was removed and a new 24-gauge catheter was inserted in the urethra for intravesical instillation of MBT-2 cells.

MBT-2 cells stably expressing luciferase (MBT-2-Luc; luciferase L4899 obtained from Sigma-Aldrich Japan G.K.) were generated by transfecting MBT-2 cells with the pGL3-Luc plasmid using a TransIT^®^-3T3 transfection kit (Mirus Bio LLC, Madison, WI, USA). Cells that stably expressed luciferase were obtained by selection with 500 μg/m1 of G418 for two weeks. Following G418 selection, growth medium from MBT-2-Luc was tested for luciferase activity to confirm the expression and secretion of luciferase into the cell medium. Luciferase-transfected MBT-2 cells (5×10^4^ cells mixed with 0.1 ml PBS) were instilled and retained for 1.5 h by stitches. Every 10 days, tumor imaging was performed following i.p. administration of luciferin using bioluminescence technology (Xenogen IVIS200 system; Xenogen Coproration, Hopkinton, MA, USA). Prior to the initiation of the treatment, the mice were randomly divided into three groups [seven mice per group; control (PBS), low-dose BCG and high-dose BCG] according to the tumor imaging results, as determined by the luciferase expression. BCG (1×10^5^ CFU/100 μl or 1×10^7^ CFU/100 μl) was administered intravesically once weekly for three weeks. The mice received i.p. injections of luciferin, and the luciferase expression in the tumors was measured by the Xenogen IVIS200 System. The body weights of the mice were measured once a week. All mice were sacrificed on day 60 after each treatment, and one mouse was selected from each group for a histological analysis. The mice were killed using CO_2_ for euthanasia and according to the guidelines for the euthanasia of animals (Edition, 2013).

### Flow cytometry

For the flow cytometry, 100-μl aliquots of blood collected from the mouse bladder cancer models were mixed with 4 μl 2 mmol/l EDTA and incubated with fluorescein isothiocyanate-labeled anti-mouse CD11b and CD4 monoclonal antibodies (mAbs) and phycoerythrin-labeled anti-mouse Gr-1 and Forkhead box P3 (Foxp3) mAbs for 1 h at 4°C, prior to washing twice with PBS. The cells were resuspended in 250 μl PBS and analyzed with a BD FACSCalibur™ flow cytometer (BD Biosciences, Bedford, MA, USA), using a lymphocyte gating strategy.

### Histology

One mouse from each group was sacrificed for histological analysis on day 60 after each treatment. The tissues were removed, fixed in formalin, embedded in paraffin and sectioned. The 5-μm sections were stained with hematoxylin and eosin and examined for histological changes using an Olympus IX71 microscope (Olympus, Tokyo, Japan).

### Statistical analysis

The data are presented as the mean ± standard error of the mean. An unpaired Student’s t-test was performed to analyze the difference between any two groups. Differences were considered to be significant if P<0.05.

## Results

### Antitumor effect of low-/high-dose BCG treatment in the C3H/HeN mouse orthotopic bladder cancer model

**Figure 1:**
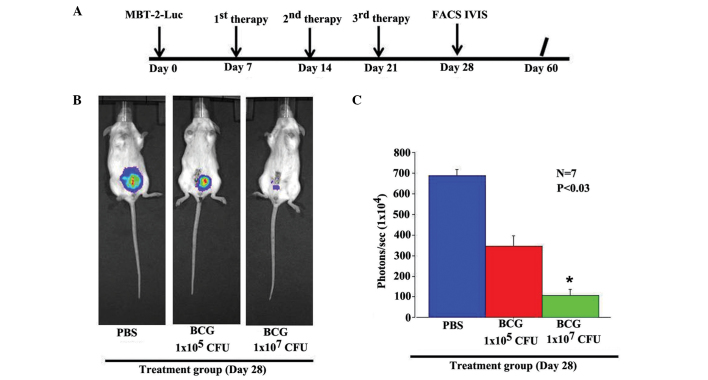
Antitumor effect of high-/low-dose BCG treatment on the growth of orthotopic bladder cancer tumors in mice. (A) Protocol and indicated time-points for the study. (B) Bioluminescence images of mice from each treatment group day 28. The animals with the tumors derived from the MBT-2 cells stably transfected with luciferase were imaged following luciferin injection via a Xenogen IVIS200 system instrument. (C) The tumor volume was analyzed by the Xenogen IVIS200 system following the indicated treatment. The tumor volume of each mouse was determined by region-of-interest analysis of total photons per second. Seven mice were analyzed in each group. ^*^A significant difference was observed in comparison with the PBS and low-dose groups. PBS, phosphate-buffered saline; BCG, Bacillus Calmette-Guérin; FACS, fluorescence-activated cell sorting.

The therapeutic efficacy of BCG on the growth of MBT-2 cells was assessed *in vivo*. Luciferase-expressing MBT-2 cells were injected orthotopically in immunocompetent mice. Mice bearing orthotopic bladder cancer were treated intravesically on days 7, 14 and 21 post-cancer implantation with PBS, low-dose BCG (1×10^5^ CFU/100 μl) or high-dose BCG (1×10^7^ CFU/100 μl) (Fig. 1A). After 60 days of treatment, the PBS-treated mice did not show a significant growth-inhibitory effect, as assessed by the Xenogen IVIS200 System (Fig. 1B and C). Low-dose BCG administration induced a 50% reduction of tumor growth. However, the high-dose BCG induced a synergistic inhibitory effect that was greater than that induced by the administration of the low-dose BCG. The differences in the levels of luciferase expression correlated with the tumor area.

### BCG affects the activation of CD11b^+^/Gr-1^+^ cells and Tregs in the peripheral blood of murine models of MBT-2 tumors

**Figure 2:**
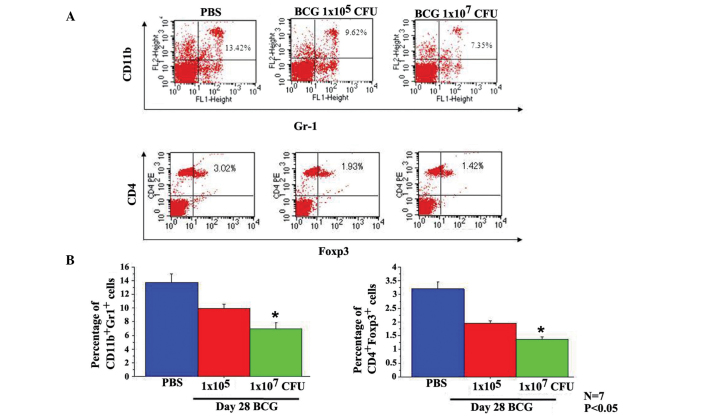
The percentage of peripheral CD11b^+^/Gr-1^+^ MDSCs and CD4^+^/Foxp3^+^ regulatory T cells in the total lymphocytes was analyzed by FACS in each mouse. (A) Representative FACS data. The blood sample was collected on day 14 after the treatment. (B) The percentage of CD11b^+^/Gr-1^+^ MDSCs and CD4^+^/Foxp3^+^ T cells in the total lymphocytes was quantified by FACS analysis and is shown in the indicated groups. Seven mice were analyzed in the treatment groups. ^*^A significant difference was observed in comparison with the PBS group. PBS, phosphate-buffered saline; BCG, Bacillus Calmette-Guérin; FACS, fluorescence-activated cell sorting; MDSC, myeloid-derived suppressor cell; CD, cluster of differentiation; Foxp3, Forkhead box P3.

**Figure 3:**
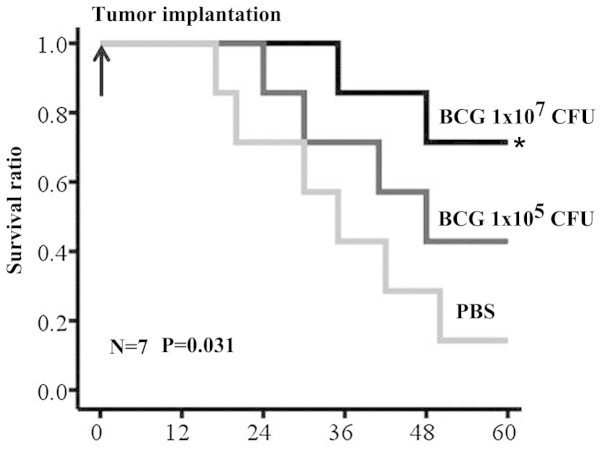
Kaplan-Meier analysis of the survival rate of mice according to intravesical instillation following MBT-2 implantation in an orthotopic bladder cancer model. ^*^A significant difference was observed in comparison with the PBS group. PBS, phosphate-buffered saline; BCG, Bacillus Calmette-Guérin.

The potential mechanisms underlying the antitumor effect elicited by low-/high-dose BCG were next explored. In this experiment, the peripheral blood samples obtained from mouse models of MBT-2 tumors were assessed. Blood was collected prior to initiating the treatment and at day 28 after the treatment, and the CD11b^+^/Gr-1^+^ cells and CD4^+^/Foxp3^+^ Tregs were quantified by fluorescence-activated cell sorting (FACS) analysis. As shown in Fig. 2A and B, the CD11b^+^/Gr-1^+^ cells and CD4^+^/Foxp3^+^ Tregs were observed in all the mice prior to treatment. After four weeks of treatment, the CD11b^+^/Gr-1^+^ cell population in the low-dose-treatment group was higher than that in the mice treated with the high-dose BCG. However, the largest CD11b^+^/Gr-1^+^ cell population was observed in the mice of the control group. The size of the population of CD4^+^/Foxp3^+^ Tregs was also different between the treatment and non-treatment groups. The largest CD4^+^/Foxp3^+^ Treg population was observed in the control group and the smallest appeared in the high-dose BCG group (Fig. 2A and B). These experiments were repeated three times, and the values shown in the figure are the average of the three experiments. At day 60 after MBT-2 implantation, all the mice in the control group had died, with the exception of one mouse. Two of the seven low-dose BCG-treated mice survived, and five of the seven mice survived in the high-dose BCG group. Significant differences in survival time existed between the control and treatment groups (Fig. 3) (P<0.05).

### Tumor-specific cytological changes

**Figure 4:**
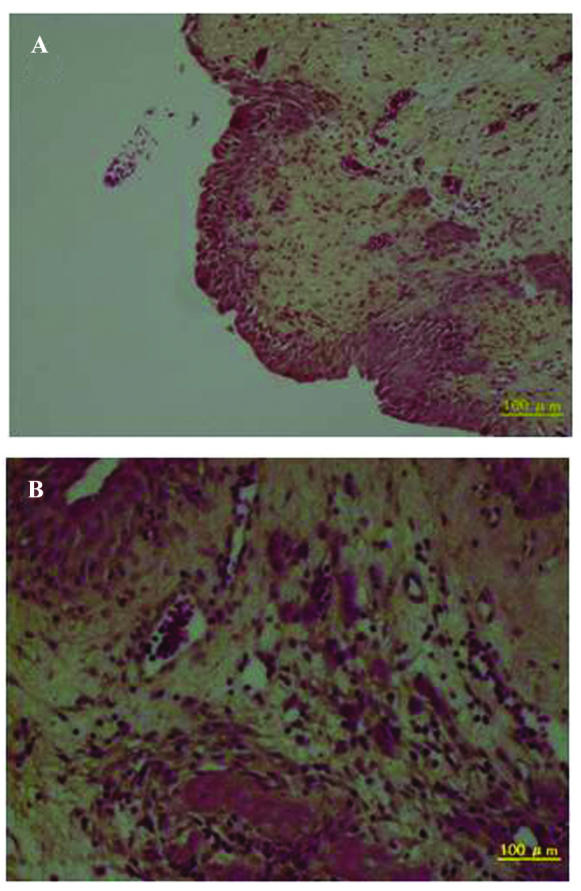
High-grade bladder cancer (hematoxylin and eosin staining) in the low-dose BCG group. (A) Magnification, ×10 and (B) magnification, ×100.

In order to investigate the mechanism and effect of BCG on bladder tumors and other body tissues, the mice were sacrificed on day 60 after treatment initiation. A histopathological analysis revealed extensive tumor tissue degeneration in the orthotopic bladder cancer of mice in the high- and low-dose BCG groups (Fig. 4A and B), but tumor tissue degeneration was not observed in the control group. These findings provided evidence for the tumor volume reduction or growth inhibition observed following each treatment. However, an analysis of liver sections from the mice treated with BCG demonstrated no histological evidence of hepatocellular damage.

## Discussion

It has been reported that ~80% of bladder cancers are superficial at the time of diagnosis, and a high rate of local recurrence and progression occurs following transurethral resection of bladder tumor (TUR-Bt) (12). The high rate of recurrence (70%) and the progression rate (45% for grade three), as well as the unpredictability of progression patterns, have led to the widespread use of intravesical adjuvant therapy following TUR-Bt. Intravesical immunotherapy with BCG is an effective adjuvant therapy for high-grade, non-muscle-invasive bladder cancers (13,14).

In the present study, it was investigated whether BCG treatment induced a change in immune cells in a bladder cancer murine model. Our data demonstrated that the population of MDSCs (CD11b^+^/Gr-1^+^) was decreased in murine models of orthotopic bladder cancer receiving BCG treatment. A dose-dependent antitumor and survival effect was shown in the orthotopic models receiving BCG treatment. High-dose BCG treatment exhibited significantly enhanced tumor inhibition and survival benefits versus the control group. Low-dose BCG did not demonstrate a significant difference in tumor inhibition and survival versus the control group.

To examine the anticancer immunomodulation in each mouse, the ratio of peripheral CD11b^+^/Gr-1^+^ MDSCs was measured by FACS analysis. The population of MDSCs was significantly downregulated following the high-dose BCG therapy compared with the low-dose therapy. Therefore, the *in vivo* synergistic effect of high-dose BCG induced the downregulation of peripheral CD11b^+^/Gr-1^+^ MDSCs that was observed in the bladder cancer orthotopic model. This immunological synergistic effect in the suppression of CD11b^+^/Gr-1^+^ MDSCs may explain the robust antitumor therapeutic effects of the high-dose BCG therapy.

Numerous studies have shown that MDSCs represent a heterogeneous population of variably matured myeloid cells, which mediate the suppression of antitumor immune responses (15,16). In mice with tumors, CD11b^+^/Gr-1^+^ MDSCs can accumulate during cancer progression and inhibit antitumor T-cell responses. Two mechanisms are used by MDSCs to downregulate the activation T cells: MDSC-mediated downregulation of L-selectin and MDSC sequestration of cysteine, an amino acid that the T cells are unable to synthesize *de novo* and that they require for activation.

In recent years, a number of bases (17) and clinical trials (18,19) have shown that BCG antigens can be presented at the cell surfaces of urothelial and antigen-presenting cells in major histocompatibility complex class II, thus stimulating CD4^+^ T cells and inducing a primarily T-helper type 1 (Th1) immune response (6,20). Within the tumor microenvironment, the immunosuppressive effects of Tregs may prevent the initiation of antitumor immune responses by interferon-γ-producing CD4^+^ Th1 and CD8^+^ T cells; however, these effects may not be capable of overcoming the already established cycle of IL-6- and signal transducer and activator of transcription 3-mediated inflammation (21). In the present study, the change in CD4^+^/Foxp3^+^ Tregs also exhibited the same result: Following the BCG treatment, the population of CD4^+^/Foxp3^+^ Tregs was decreased in the blood. These findings may assist in the clarification of the mechanism underlying the synergistic antitumor immunological response elicited by BCG.

In conclusion, the present study in an orthotopic mice bladder cancer model indicates that the antitumor and tumor-immunology efficacy of BCG is dose-dependent. The activation of MDSCs also suggests dose-dependence. These findings are notable in terms of the clinical evaluation of this therapy for patients with bladder cancer. The outcomes of this study also provide important implications regarding antitumor immune responses in human cancer.
